# Safety and Efficacy of Avaren-Fc Lectibody Targeting HCV High-Mannose Glycans in a Human Liver Chimeric Mouse Model

**DOI:** 10.1016/j.jcmgh.2020.08.009

**Published:** 2020-08-27

**Authors:** Matthew Dent, Krystal Hamorsky, Thibaut Vausselin, Jean Dubuisson, Yoshinari Miyata, Yoshio Morikawa, Nobuyuki Matoba

**Affiliations:** 1 Department of Pharmacology and Toxicology; 2 Department of Medicine; 3 James Graham Brown Cancer Center; 4 Center for Predictive Medicine, University of Louisville School of Medicine, Louisville, Kentucky; 5 University of Lille, Centre national de la recherche scientifique, INSERM, Centre Hospitalier Universitaire Lille, Institut Pasteur de Lille, U1019, UMR 8204, Center for Infection and Immunity of Lille, Lille, France; 6 PhoenixBio USA Corporation, New York, New York

**Keywords:** Hepatitis C Virus, Entry Inhibitor, Plant-Made Pharmaceutical, High-Mannose Glycan, Antiviral Therapy, ALT, alanine aminotransferase, AvFc, Avaren-Fc, DAA, direct-acting antiviral, h-Alb, human albumin, HCV, hepatitis C virus, HCVcc, cell-culture-derived hepatitis C virus, HCVpp, hepatitis C virus pseudovirus, HIV, human immunodeficiency virus, HMG, high-mannose glycans, PBS, phosphate-buffered saline, RT-PCR, reverse-transcription polymerase chain reaction, uPA/SCID, urokinase plasminogen activator/severe combined immunodeficiency

## Abstract

**Background & Aims:**

Infection with hepatitis C virus (HCV) remains a major cause of morbidity and mortality worldwide despite the recent advent of highly effective direct-acting antivirals. The envelope glycoproteins of HCV are heavily glycosylated with a high proportion of high-mannose glycans (HMGs), which serve as a shield against neutralizing antibodies and assist in the interaction with cell-entry receptors. However, there is no approved therapeutic targeting this potentially druggable biomarker.

**Methods:**

The anti-HCV activity of a fusion protein consisting of Avaren lectin and the fragment crystallizable (Fc) region of a human immunoglobulin G1 antibody, Avaren-Fc (AvFc) was evaluated through the use of in vitro neutralization assays as well as an in vivo challenge in a chimeric human liver (PXB) mouse model. Drug toxicity was assessed by histopathology, serum alanine aminotransferase, and mouse body weights.

**Results:**

AvFc was capable of neutralizing cell culture–derived HCV in a genotype-independent manner, with 50% inhibitory concentration values in the low nanomolar range. Systemic administration of AvFc in a histidine-based buffer was well tolerated; after 11 doses every other day at 25 mg/kg there were no significant changes in body or liver weights or in blood human albumin or serum alanine aminotransferase activity. Gross necropsy and liver pathology confirmed the lack of toxicity. This regimen successfully prevented genotype 1a HCV infection in all animals, although an AvFc mutant lacking HMG binding activity failed.

**Conclusions:**

These results suggest that targeting envelope HMGs is a promising therapeutic approach against HCV infection, and AvFc may provide a safe and efficacious means to prevent recurrent infection upon liver transplantation in HCV-related end-stage liver disease patients.

## Graphical abstract

**Figure:**
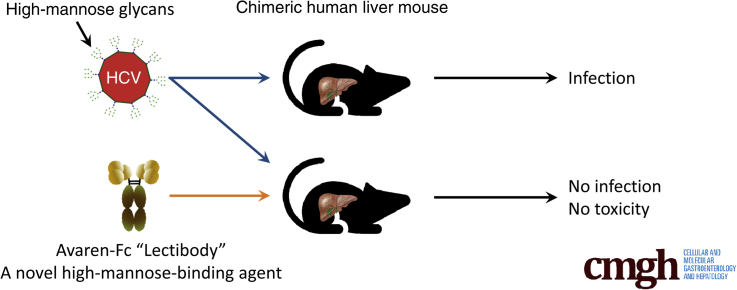

**Summary**

Hepatitis C virus (HCV) infection remains a major cause of end-stage liver disease. Here, we show the efficacy and safety of a novel biotherapeutic targeting a HCV glycobiomarker in a mouse model, providing a foundation for a new anti-HCV strategy.

Hepatitis C virus (HCV) is an enveloped monopartite positive-sense single-stranded RNA virus in the family *Flaviviridae* and the causative agent of hepatitis C disease. Its genome encodes 3 structural (core, E1, E2) and 7 nonstructural proteins (p7, NS2, NS3, NS4A, NS4B, NS5A, and NS5B).^1^ HCV is highly heterogenous and distributed globally, consisting of 7 genotypes, each subdivided further into multiple subtypes. Genotypes 1 and 2 are the predominant genotypes worldwide and are particularly concentrated in high-income and upper-middle-income countries, whereas genotypes 3 and 4 are more common in lower-middle and low-income countries.^2^ In the United States, injection drug use represents the primary risk factor for contracting HCV infection.^3^^,^^4^ Approximately 15%–25% of people acutely infected with HCV will clear the virus, while the remainder will develop chronic infection that can persist largely unnoticed for decades. Indeed, many HCV carriers discover their chronic infection after they have developed cirrhosis.^5^ Chronic HCV infection also is associated with the development of hepatocellular carcinoma, and patients with the disease are more likely to develop cryoglobulinemia and non-Hodgkin’s lymphoma.^6^

There is no vaccine currently available for HCV. Before 2011, the standard chronic HCV treatment was a nonspecific antiviral medication using ribavirin combined with pegylated interferon-α, which was associated with significant toxicity and limited treatment efficacy.^7^ In 2011, the US Food and Drug Administration approved the first-generation of direct-acting antivirals (DAAs) for HCV: boceprevir and telaprevir, both of which inhibit the viral protease (NS3/4A), but required co-treatment with ribavirin and peginterferon.^8^^,^^9^ Further approval of more potent DAAs, such as NS3/4A, NS5B, and NS5A inhibitors, led to the development of oral ribavirin/peginterferon-free regimens.^5^ Multi-DAA regimens achieve sustained virologic response (defined as a period of time with no viral RNA detection) rates as high as 100%, and are less toxic and more tolerable than their predecessors.^10^, ^11^, ^12^, ^13^ Although the cure rates are remarkable, populations of patients exist who may not benefit from DAA therapy,^14^ especially patients with decompensated cirrhosis resulting from chronic HCV infection, for whom liver transplantation may be a last resort.^15^ Moreover, recurrent infection occurs universally and rapidly after liver transplantation,^16^^,^^17^ which increases the risk of accelerated cirrhosis, graft failure, and death.^18^ DAAs, by their nature, cannot prevent recurrent infection. Therefore, alternative or complementary therapies to DAAs that can block viral entry to target cells, such as antibodies or other molecules acting alike, may need to be considered in these circumstances.^18^^,^^19^ However, there is currently no entry inhibitor approved for HCV treatment.

The HCV envelope proteins E1 and E2 are heavily glycosylated and, similar to glycoproteins of other enveloped viruses (eg, human immunodeficiency virus [HIV] and the coronaviruses), have a high proportion of high-mannose-type *N*-glycans (HMGs) on their surface.^20^, ^21^, ^22^ These glycans typically are processed to hybrid and complex forms on glycoproteins secreted by healthy cells.^23^ Thus, the HMGs on the surface of HCV may be considered a druggable target. We previously described the development of a fusion protein consisting of HMG-targeting Avaren lectin and the fragment crystallizable (Fc) region of a human immunoglobulin G1 antibody, or *lectibody*, called Avaren-Fc (AvFc), which was shown to bind with high affinity to clusters of HMGs on the HIV envelope protein glycoprotein (gp)120 and effectively neutralize multiple HIV clades and groups including HIV-2 and simian immunodeficiency virus.^24^ Further analysis indicated that AvFc can bind to HCV E2 protein.^24^ Therefore, in this study, we aim to investigate the anti-HCV therapeutic potential of AvFc in in vitro neutralization assays and an in vivo HCV challenge study using PXB mice (PhoenixBio Co., Japan), a chimeric urokinase plasminogen activator/severe combined immunodeficiency (uPA/SCID) mouse model transplanted with human hepatocytes (reviewed by Tateno and Kojima^25^).

## Results

### AvFc Shows Broad Anti-HCV Activity In Vitro

**Figure 1:**
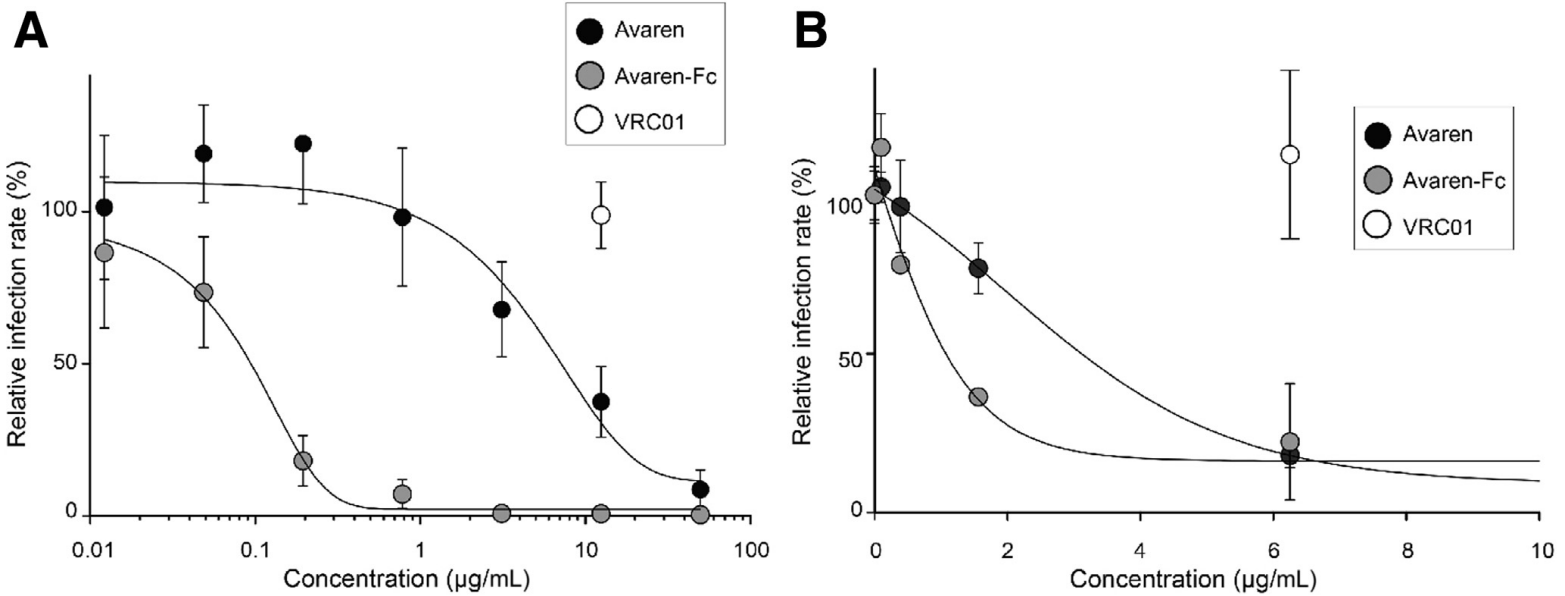
**In vitro HCV inhibition assays.** (*A*) Avaren lectin and AvFc inhibit cell–culture derived HCV. The JFH1 virus was preincubated with Avaren lectin, AvFc, or the control antibody VRC01 for 30 minutes at 37°C before incubation with Huh-7 cells. At 48 hours after infection, infected cells were quantified by indirect immunofluorescence with an HCV-specific antibody. Results are expressed as a percentage of infection compared with a control infection in the absence of compound. Error bars indicate SEM values from at least 3 independent experiments. (*B*) Avaren lectin and AvFc inhibit HCV entry. Retroviral pseudotypes bearing HCV envelope glycoproteins of the JFH1 virus (HCVpp) were preincubated with Avaren lectin, AvFc, or the control antibody VRC01 for 30 minutes at 37°C before incubation with Huh-7 cells. At 48 hours after infection, cells were lysed to quantify the luciferase activity. Results are expressed as the percentage of infection compared with the control infection in the absence of compound. Error bars indicate SEM values from at least 3 independent experiments.

Building on our previous observation that AvFc has affinity to a recombinant HCV E2 envelope protein,^24^ we first examined whether AvFc inhibits HCV infection in vitro using multiple genotypes of cell culture-produced virus (HCVcc) or pseudotyped virus (HCVpp). AvFc significantly blocked the infection of the human liver cell line Huh-7 by HCVcc from genotypes 1a, 2a, 4a, 5a, and 6a, with 50% inhibitory concentration values in the low nanomolar range (Table 1 and Figure 1*A*). Compared with Avaren lectin monomer, AvFc overall showed approximately 2-log higher activity, although no inhibitory effect was observed for the plant-produced anti-HIV broadly neutralizing antibody VRC01, which shares the same human IgG1 Fc region with AvFc.^26^ In addition, Avaren lectin and AvFc, but not VRC01, effectively neutralized HCVpp harboring a murine leukemia virus backbone, suggesting that the lectin and the lectibody act as an entry inhibitor (Figure 1*B*).

**Table 1:** IC_50_ Values for AvFc and Avaren Against HCVcc

Virus	Genotype	Avaren lectin IC_50_, *nmol/L*	AvFc IC_50_, *nmol/L*
JFH1/H77	1a	529.28 ± 158.78	1.69 ± 0.39
JFH1	2a	484.62 ± 109.16	1.69 ± 0.78
JFH1/ED43	4a	204.27 ± 1.65	2.85 ± 0.91
JFH1/SA13	5a	148.86 ± 2.48	2.33 ± 0.13
JFH1/HK6a	6a	114.95 ± 52.93	1.95 ± 0.78
	Average	269.39 ± 65.00	2.10 ± 0.60

IC_50_, 50% inhibitory concentration.

### Formulation of AvFc Into a Biocompatible Buffer for In Vivo Studies

**Figure 2:**
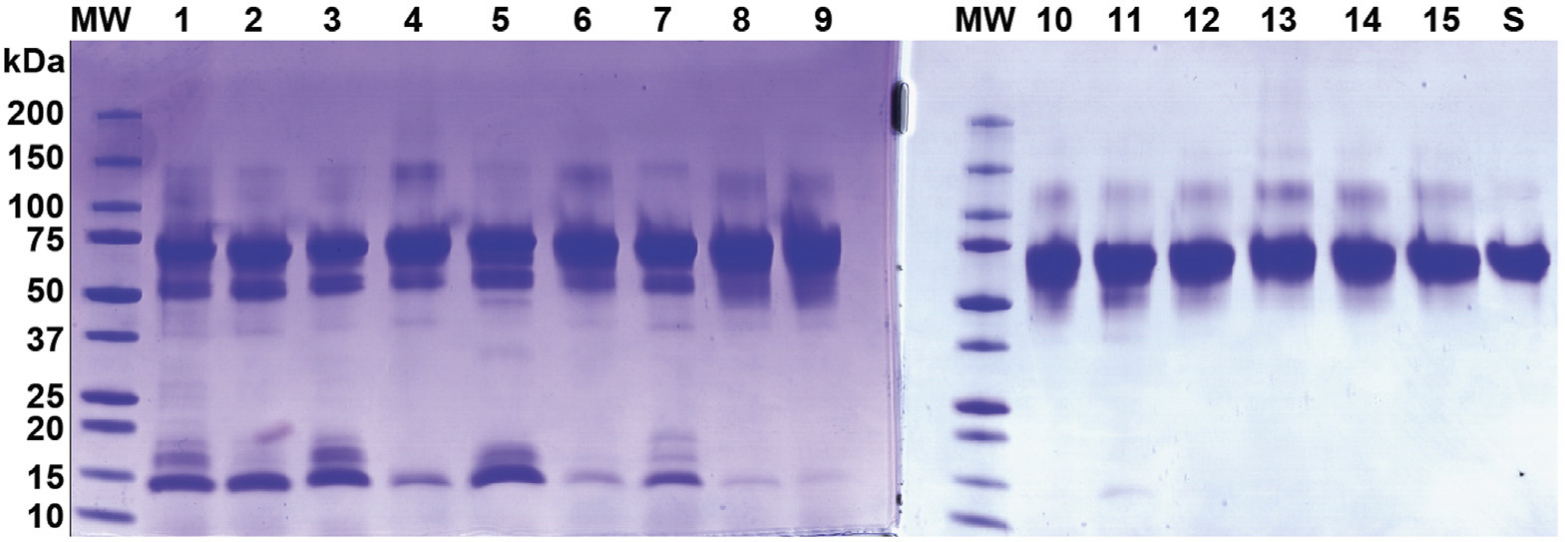
**Stability of AvFc in various buffers.** The initial buffer screening was performed by incubating 1 mg/mL of AvFc at 37°C for 2 weeks in various buffers without any excipient (listed in Table 2), followed by sodium dodecyl sulfate–polyacrylamide gel electrophoresis analysis. The image shows a Coomassie Brilliant Blue–stained gel resolving 10 μg of AvFc from respective buffers, including glutamate at pH 4.5 (*lane 1*) and 5.0 (*lane 2*); acetate at pH 4.5 (*lane 3*) and 5.5 (*lane 4*); citrate at pH 5.0 (*lane 5*) and 6.0 (*lane 6*); succinate at pH 5.5 (*lane 7*) and 6.5 (*lane 8*); histidine at pH 6.0 (*lane 9*) and 7.0 (*lane 10*); phosphate at pH 6.5 (*lane 11*), 7.0 (*lane 12*), and 7.5 (*lane 13*); Tris at pH 7.5 (*lane 14*); and PBS (*lane 15*). At pH 6.0 and less (buffers 1–9), AvFc showed significant degradation after 2 weeks at 37°C. AvFc did not significantly degrade in buffers 10–15, and therefore these were chosen for further preformulation analysis. MW, molecular weight marker; S, standard AvFc control.

**Figure 3:**
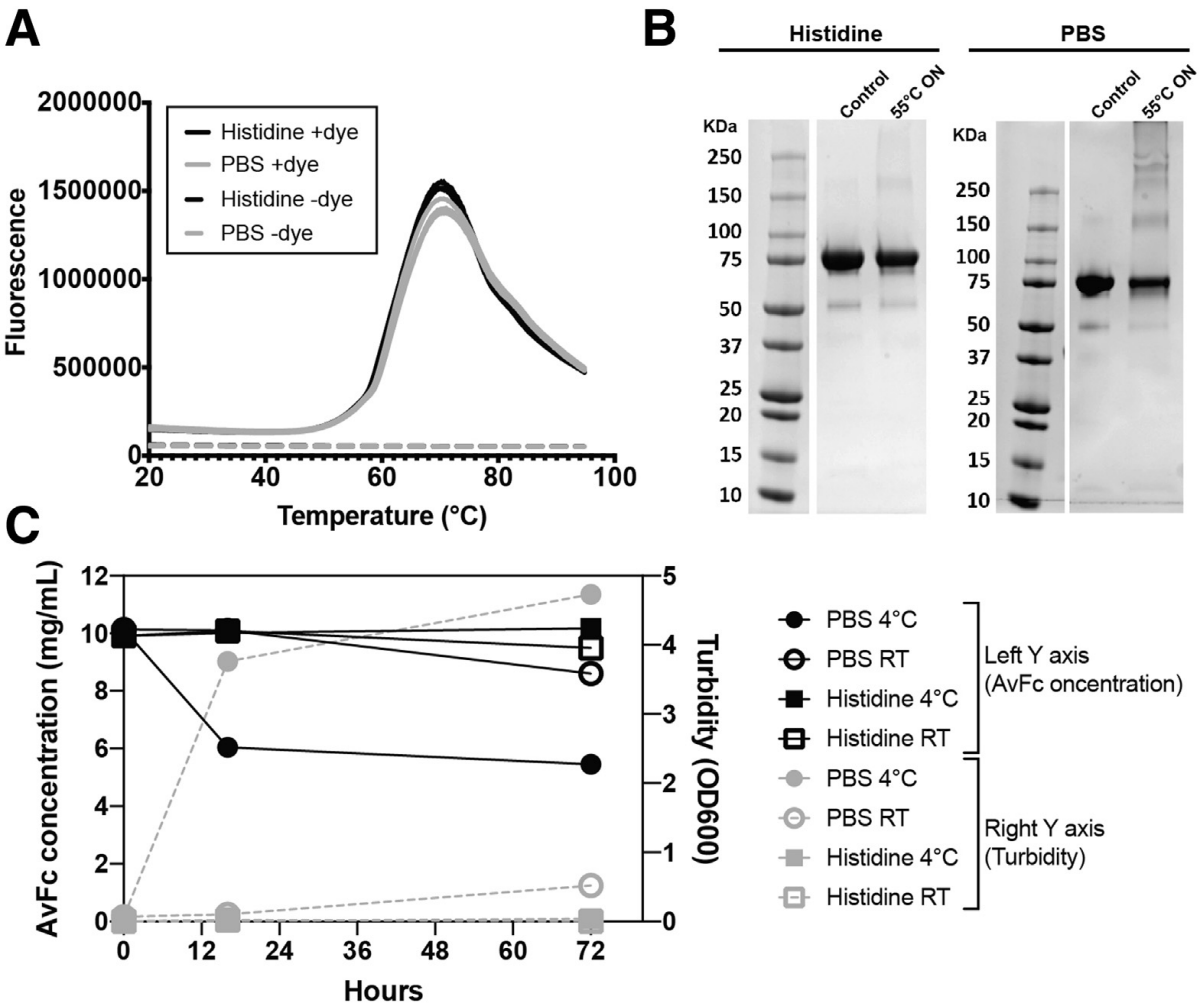
**Liquid formulation development for AvFc.** (*A*) Differential scanning fluorimetry for melting temperature measurement. AvFc was prepared in 30 mmol/L histidine buffer, 100 mmol/L NaCl, 100 mmol/L sucrose (histidine, *black line*), or PBS (*grey line*) at a concentration of 1 mg/mL and analyzed in triplicate in the presence (*solid line*) or absence (*dashed line*) of the fluorescent dye SYPRO Orange (ThermoFisher Scientific, Waltham, MA). Melting temperature values were 62.49°C ± 0.13°C in the histidine buffer and 62.68°C ± 0.25°C in PBS, as determined by the vertex of the first derivative of the relative fluorescence unit values. (*B*) Accelerated stability testing of AvFc in the histidine buffer and PBS. AvFc, prepared at 1 mg/mL in the histidine buffer or PBS were incubated overnight at 55°C, and 10 μg of the protein from each formulation was analyzed by sodium dodecyl sulfate–polyacrylamide gel electrophoresis under nonreducing conditions. A representative Coomassie-stained gel image is shown. The band at around 75 kilodaltons corresponds to AvFc. Note that after overnight incubation, PBS shows less band intensity for AvFc and more large-size aggregate bands than the histidine buffer. (*C*) Time course of concentration change and the turbidity of AvFc solution in the histidine buffer and PBS. AvFc was formulated at 10 mg/mL in respective buffers and incubated at 4°C or room temperature (RT). After 16 and 72 hours, the concentration was measured using a theoretical extinction coefficient at 280 nm of 1.6493 (mg/mL)^-1^ cm^-1^, whereas turbidity was assessed by absorbance at 600 nm. Representative data are shown for samples analyzed in triplicate.

Previously, we found that AvFc has limited solubility in phosphate-buffered saline (PBS) at concentrations greater than 1 mg/mL (unpublished observation). To facilitate in vivo studies, we screened for an optimal liquid formulation for systemic administration that can impart improved stability and solubility to AvFc at higher concentrations. Initial buffer screening showed that AvFc is prone to degradation at and below a pH of 6.5, suggesting that AvFc is not stable in acidic pH conditions (Figure 2, Table 2). Further preformulation studies led us to identify an optimal buffer composed of 30 mmol/L histidine, pH 7.0, 100 mmol/L sucrose, and 100 mmol/L NaCl. Although AvFc showed comparable melting temperature in the histidine buffer and PBS in differential scanning fluorimetry (62.49°C ± 0.13°C vs 62.68°C ± 0.25°C) (Figure 3*A*), sodium dodecyl sulfate–polyacrylamide gel electrophoresis analysis showed that the lectibody holds superior stability in the histidine buffer upon accelerated stability testing via overnight incubation at 55°C (Figure 3*B*). When concentrated to approximately 10 mg/mL, AvFc remained stable in solution in the histidine buffer over 72 hours at 4°C and room temperature, while showed a significant concentration decrease concomitant with increasing turbidity in PBS (Figure 3*C*), further showing the histidine buffer’s superiority for AvFc formulation.

**Table 2:** Buffers Used in the Initial Screening of AvFc Preformulation Analysis

Number	Formulation	pH
1	30 mmol/L glutamate (5.61 g/L NaOOCCH_2_CH_2_CH(NH_2_)COOH × H_2_O)^a^	4.5
2	30 mmol/L glutamate (5.61 g/L NaOOCCH_2_CH_2_CH(NH_2_)COOH × H_2_O)^a^	5.0
3	30 mmol/L acetate (2.46 g/L CH_3_COONa)^a^	4.5
4	30 mmol/L acetate (2.46 g/L CH_3_COONa)^a^	5.5
5	30 mmol/L citrate (350 mL 0.1 mol/L C_6_H_8_O7 × H_2_O, 650 mL 0.1 mol/L C_6_H_5_O_7_Na_3_ × 2 H_2_O)	5.0
6	30 mmol/L citrate (115 mL 0.1 mol/L C_6_H_8_O7 × H_2_O, 885 mL 0.1 mol/L C_6_H_5_O_7_Na_3_ × 2 H_2_O)	6.0
7	30 mmol/L succinate (4.86 g/L NaOOCCH_2_CH_2_COONa)^a^	5.5
8	30 mmol/L succinate (4.86 g/L NaOOCCH_2_CH_2_COONa)^a^	6.5
9	30 mmol/L histidine (4.65 g/L C_6_H_9_N_3_O_2_)^a^	6.0
10	30 mmol/L histidine (4.65 g/L C_6_H_9_N_3_O_2_)^a^	7.0
11	30 mmol/L phosphate (2.89 g/L NaH_2_PO_4_ × H_2_O, 2.42 g Na_2_HPO_4_ × 7 H_2_O)	6.5
12	30 mmol/L phosphate (1.75 g/L NaH_2_PO_4_ × H_2_O, 4.64 g Na_2_HPO_4_ × 7 H_2_O)	7.0
13	30 mmol/L phosphate (0.78 g/L NaH_2_PO_4_ × H_2_O, 6.53 g Na_2_HPO_4_ × 7 H_2_O)	7.5
14	30 mmol/L Tris (3.63 g/L NH_2_C(CH_2_OH)_3_)^a^	7.5
15	PBS (0.144 g/L KH_2_PO_4_, 9 g/L NaCl, 0.795 g/L Na_2_HPO_4_)	7.2

a pH was adjusted with 1 mol/L NaOH or 1 mol/L HCl.

### Pharmacologic and Toxicologic Analysis of AvFc in Mice

**Figure 4:**
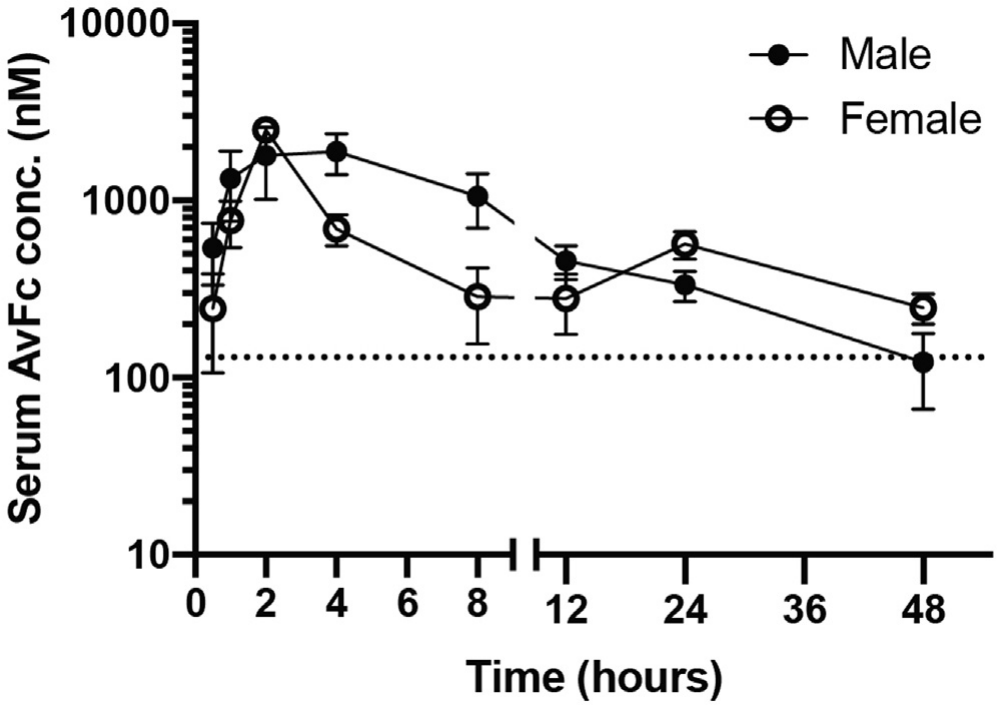
**Pharmacokinetics of AvFc in mice.** AvFc pharmacokinetics were evaluated in C57bl/6 mice after a single intraperitoneal injection of 25 mg/kg with blood sampled at various time points. Data are expressed as means ± SEM from 4 mice per group. The average half-life was 24.5 and 18.5 hours in male and female mice, respectively, as determined by the PKSolver Microsoft Excel Add-on. The peak concentration occurred between 2 and 4 hours after administration. The target trough concentration of 130 nmol/L (corresponding to 10 μg/mL) is indicated by a *dashed line*.

To determine an optimal dosing regimen for an HCV challenge experiment, a pharmacokinetic analysis of AvFc was conducted in C57bl/6 mice. After a single intraperitoneal injection of AvFc at a dose of 25 mg/kg, a peak drug concentration was observed between 2 and 4 hours, with a half-life of 24.5 hours in male animals and 18.5 hours in female animals (Figure 4). After 48 hours, in both male and female animals, the plasma concentration of AvFc remained above a target trough concentration of 130 nmol/L (10 μg/mL), at which time AvFc showed more than 90% neutralization effects against HCV (Figure 1). Consequently, these results suggested that administration of the drug every other day might be sufficient to keep the virus under control in a murine HCV challenge model.

**Figure 5:**
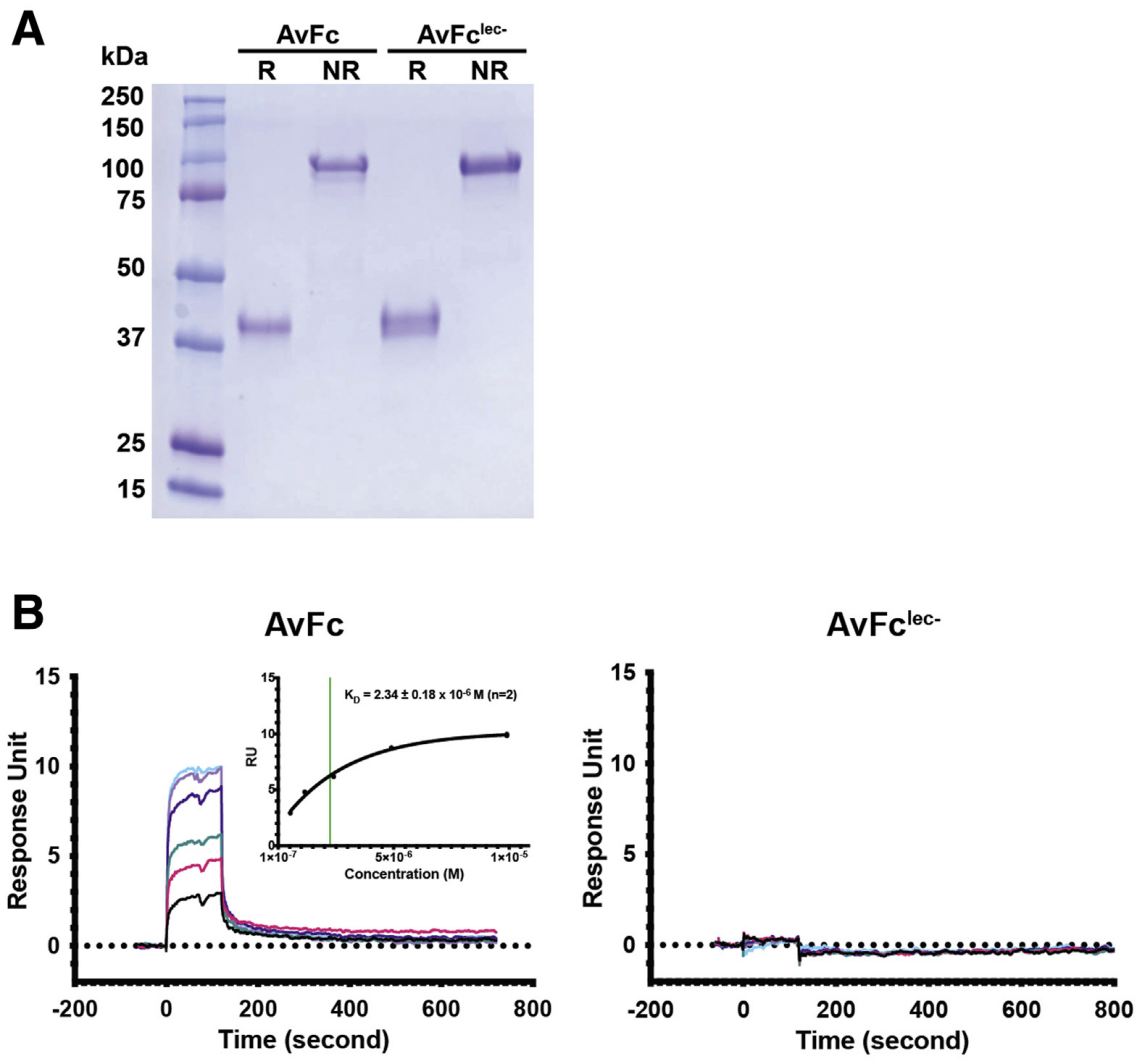
**Characterization of the non–sugar-binding mutant AvFc**^**lec-**^**.** A variant of AvFc that does not bind to HMGs was generated by mutating a tyrosine residue in each of the 3 binding pockets of Avaren (Dent et al, unpublished data). (*A*) Sodium dodecyl sulfate–polyacrylamide gel electrophoresis showing purified AvFc and AvFc^lec-^ under reducing (*R*) and nonreducing (*NR*) conditions. Under R conditions, AvFc monomer is seen at 38.5 kilodaltons; under NR conditions, AvFc dimer (via interpolypeptide disulfide bonds in the Fc region) appears at 77 kilodaltons. (*B*) Surface plasmon resonance analysis of HCV E2-binding affinity of AvFc and AvFc^lec-^. A recombinant E2 protein (Immune Technology Corp, New York, NY) was immobilized to a CM5 chip using amine coupling to a surface density of approximately 200 response units (RU). AvFc or AvFc^lec-^ then was injected over the chip surface at a rate of 30 μL/min for 120 seconds, followed by a 600-second dissociation period, with concentrations ranging from 10 to 0.625 μmol/L. Binding affinity was calculated using steady-state analysis and was determined to be 2.34 ± 0.18 × 10^-6^ mol/L (2.34 ± 0.18 μmol/L) for AvFc. Binding affinity could not be determined for AvFc^lec-^ because of the lack of measurable binding.

**Figure 6:**
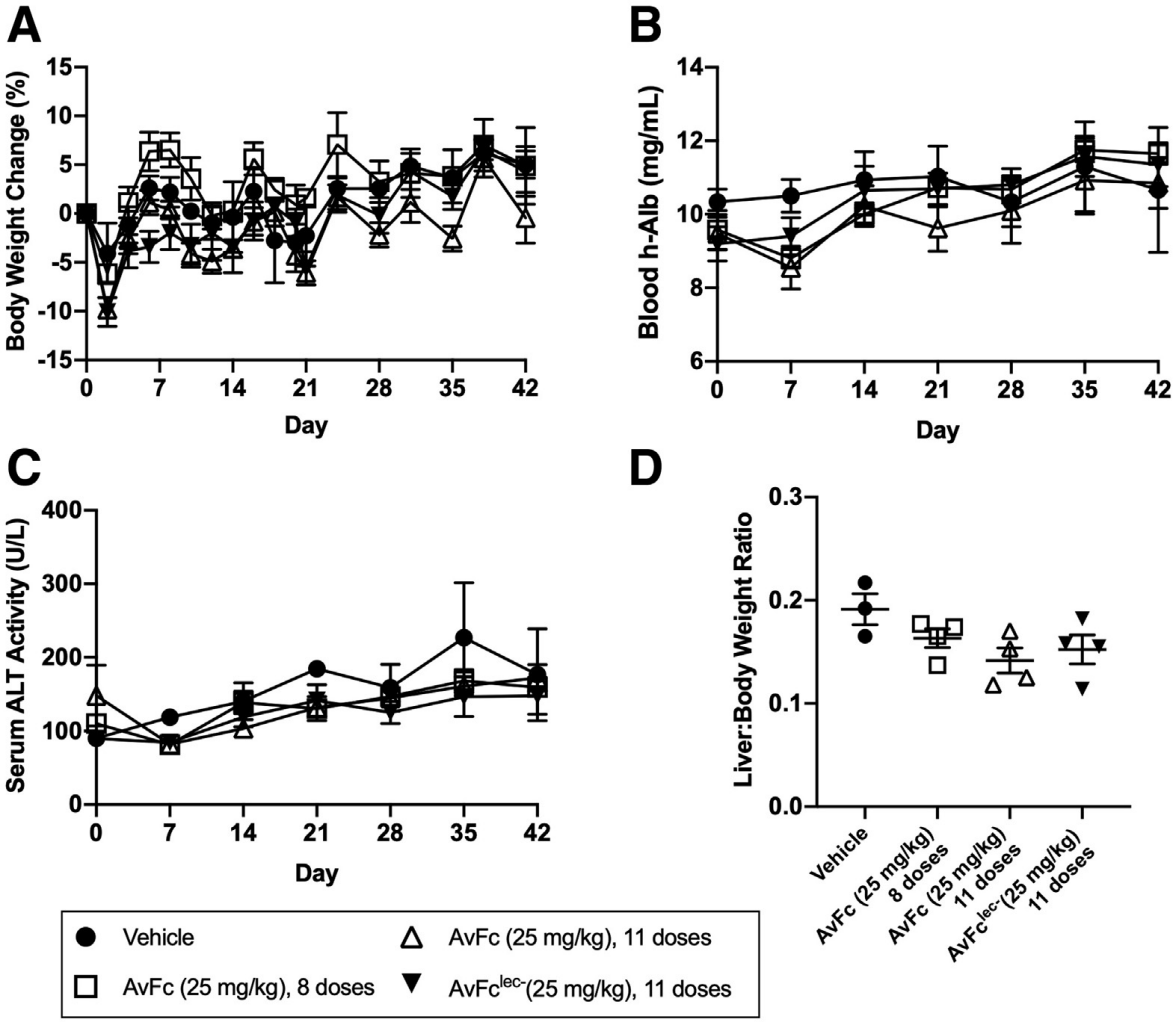
**Toxicologic analysis of systemically administered AvFc in the PXB human liver chimeric mouse model.** PXB mice were administered AvFc or AvFc^lec-^ intraperitoneally at 25 mg/kg (n = 4 each), or the histidine buffer vehicle control (n = 3) every 2 days and monitored for body weight, blood h-Alb level, and serum alanine ALT level over 42 days. (*A*) Percentage change of body weights from the initial day of dosing (day 0). (*B*) Blood h-Alb levels. (*C*) Serum ALT levels. (*D*) Ratio of liver weight to body weight of individual mice at necropsy. (*A–C*) Each data point represents means ± SEM and (*D*) individual data with means ± SEM in each group. No significant changes in any of the safety end points were noted between the groups (*A–C*, 2-way analysis of variance; *D*, 1-way analysis of variance).

We then assessed the safety of every-other-day administration of AvFc in PXB mice. To effectively discern potential toxicity associated with AvFc HMG-binding activity, we included an AvFc variant lacking HMG-binding activity as a control (AvFc^lec-^) (Figure 5*A* and *B*). PXB mice received either the vehicle (the histidine buffer described earlier) every other day for 11 total doses, AvFc at 25 mg/kg every other day for a total of 8 or 11 doses, or AvFc^lec-^ at 25 mg/kg every other day for 11 total doses. As shown in Figure 6*A–C*, no significant differences in either body weights, blood human albumin (h-Alb) levels, or serum alanine aminotransferase (ALT) activity were observed. In addition, no significant differences in relative liver weight were seen (Figure 6*D*). These results indicate that AvFc, formulated in the histidine buffer, was well tolerated in the immunocompromised mice engrafted with human hepatocytes.

**Figure 7:**
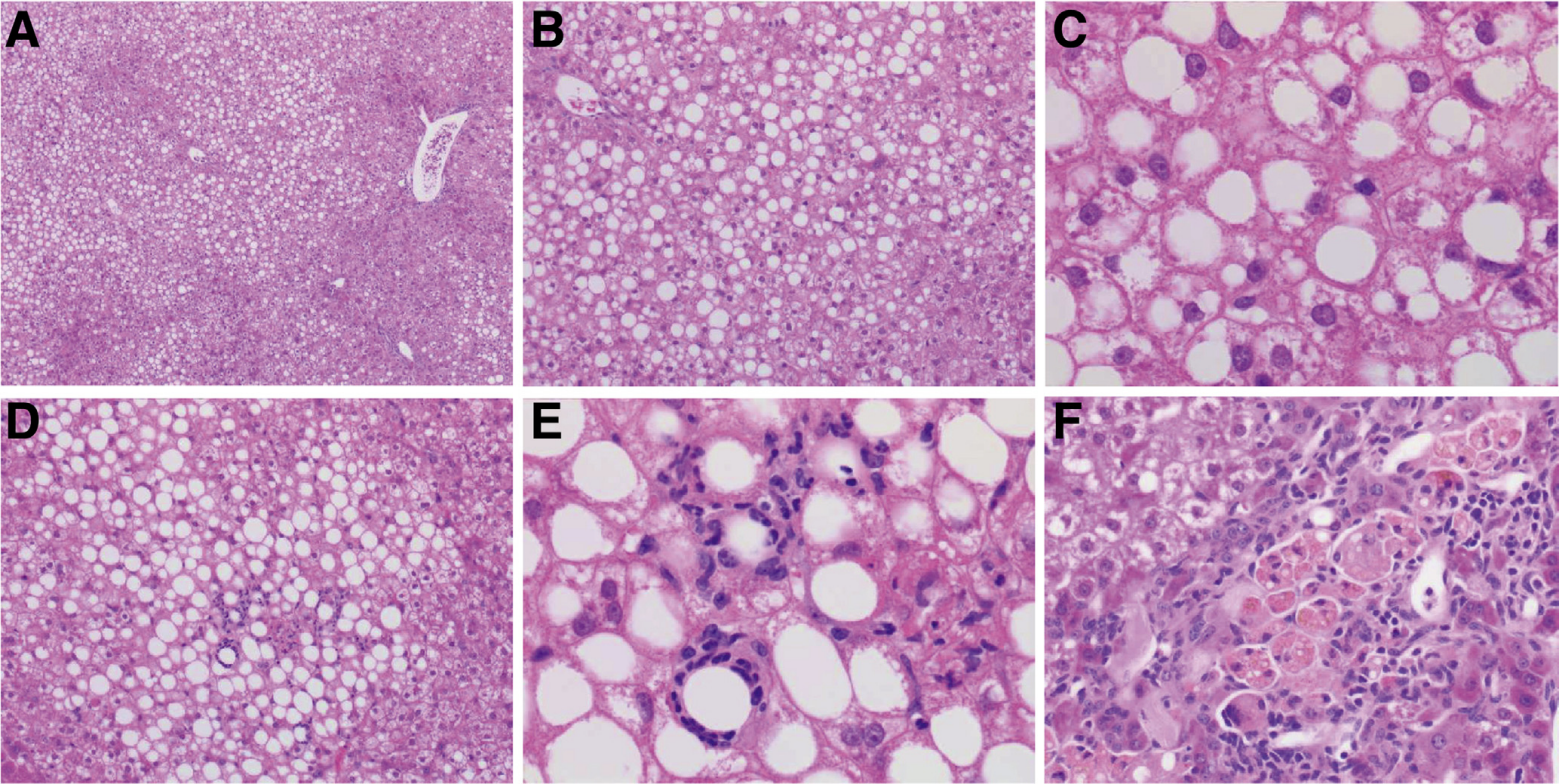
**Histopathologic examination of PXB mouse liver tissues.** Representative H&E-stained liver tissue section images corresponding to histopathologic findings in Table 2 are shown. Liver tissues are from the toxicologic study in Figure 4. (*A*) A 4× image from an animal in the vehicle control group (mouse ID: 103 in Table 2) showing low magnification of vacuolated hepatocytes. (*B*) A 10× image from a portion of panel *A*, containing many human hepatocytes with a large, well-defined, rounded vacuole. (*C*) Higher magnification (40×) of panel *B*. (*D*) A 10× image from an animal in the AvFc^lec-^ group (ID: 202 in Table 2), showing small foci of inflammatory cell inflammation in the human hepatocyte area. (*E*) Higher magnification (40×) of panel *D*. Inflammatory cells appear to surround vacuolated hepatocytes. (*F*) A 20× image from an animal in the AvFc group (8 total doses; ID: 401 in Table 2). Histiocytic brown pigmentation in the Glisson sheath was noted only in this mouse.

Histopathology was performed to evaluate any potential toxicity to the human liver grafts resulting from AvFc administration (Table 3 and Figure 7). In the human hepatocyte area, a slight to moderate (scores of 2–3 in Table 2) macrovesicular fatty change, a characteristic change of human hepatocytes in the PXB mouse, was observed in all mice, including the vehicle-treated group (Figure 7*A–C*). Minimal inflammatory cell infiltration around vacuolated hepatocytes (score, 1) was seen in 1 mouse each from the 11-dose AvFc and AvFc^lec-^ groups (Figure 7*D* and *E*); however, this was unlikely treatment-related because a similar change is seen occasionally in PXB mice (PhoenixBio, New York, NY) (unpublished observation). No AvFc treatment-specific change was observed, except for an incidental build-up of pigmentation found in the Glisson’s sheath in the liver of 1 mouse (Figure 7*F*). Collectively, it was concluded that there was no treatment-related adverse effect in the liver tissue. The full pathology report may be found in the Supplementary Information.

**Table 3:** Histopathology of Chimeric Mouse Liver Tissue

	Vehicle			AvFc^lec-^				AvFc, 11 doses				AvFc, 8 doses			
	101	102	103	201	202	203	204	301	302	303	304	401	402	403	404
Mouse hepatocytes	0	0	0	0	0	0	0	0	0	0	0	0	0	0	0
Human hepatocytesFatty change, macrovesicular	2	3	3	3	3	3	3	3	3	3	3	3	3	3	3
Infiltrate, inflammatory cell, around vacuolated hepatocyte	0	0	0	0	1	0	0	0	1	0	0	0	0	1	0
Portal canal and others															
Hepatocellular carcinoma, trabecular, with extramedullary hematopoiesis	P	0	0	0	0	0	0	0	0	0	0	0	0	0	0
Metaplasia, osseous	0	2	0	0	0	0	0	0	0	0	0	0	0	0	0
Pigmentation, brown, histiocyte, Glisson sheath, focal	0	0	0	0	0	0	0	0	0	0	0	1	0	0	0

NOTE. Numbers shown are the severity score on a scale of 0–5.

P, present.

### AvFc Protects Against HCV Infection In Vivo

**Figure 8:**
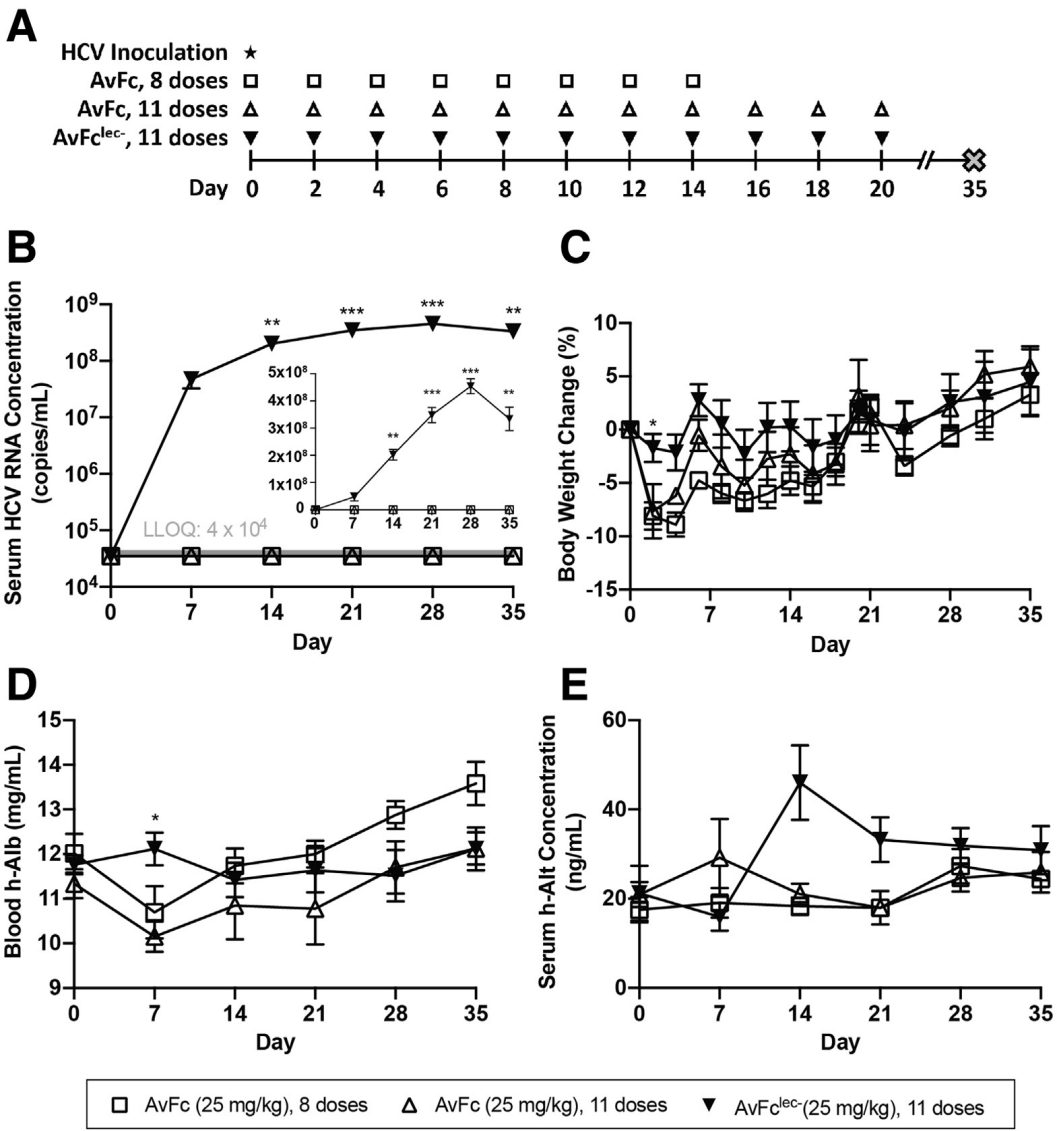
**The protective effect of AvFc against HCV challenge in PXB mice.** (*A*) Study design. PXB mice were challenged intraperitoneally with a HCV genotype 1a virus on day 0, simultaneously with an initial treatment intraperitoneally with either 25 mg/kg of AvFc or AvFc^lec-^. Treatment was continued every other day for a total of 8 or 11 doses for AvFc and 11 doses for AvFc^lec-^ (n = 5 each). The general conditions and body weights of the animals were monitored every other day, while serum HCV RNA and blood h-Alb levels were measured every 7 days. (*B*) Serum HCV RNA levels. AvFc treatment (both 8 and 11 doses) showed no detectable HCV RNA at any time point. The *gray line* indicates the lower limit of quantification, which was 4 × 10^4^ copies/mL in this assay. ∗∗*P* < .01, ∗∗∗*P* < .001 (AvFc^lec-^ vs both AvFc 8 and 11 doses); 2-way analysis of variance with the Tukey multiple comparison test. *Inset*: The graph shows the same data with the y-axis on a linear scale. (*C–E*) Time course of body weight change from day 0 (*C*), blood h-Alb levels (*D*), and serum h-Alt concentrations (*E*). Each data point represents means ± SEM in each group. ∗*P* < 0.05 (*C*, AvFc^lec-^ vs AvFc 8 doses; *D*, AvFc^lec-^ vs AvFc 11 doses; 2-way analysis of variance with the Tukey multiple comparison test. (*E*) No significant difference between groups at any time point was noted.

Lastly, we assessed the protective efficacy of AvFc against HCV infection in vivo using the treatment regimen described earlier. PXB mice were inoculated intraperitoneally with a genotype 1a virus along with initial treatment with 25 mg/kg of AvFc or AvFc^lec-^ on day 0. As shown in Figure 8*A*, AvFc^lec-^-treated mice showed high serum HCV RNA levels from day 7 after challenge through the end of the study on day 35. In sharp contrast, animals treated with both 8 and 11 doses of AvFc did not show any quantifiable level (4.0 × 10^4^ copies/mL) of HCV RNA in sera, indicating that the lectibody prevented the infection of human liver grafts by the virus. Similar to the results in Figure 3, overall no major toxicity signal was noted in body weights, h-Alb, or human ALT (h-ALT) levels between the test groups, although there was a temporal decrease in body weight and h-Alb in 1 of the AvFc-treated groups at an early time point, indicating that the liver grafts remained functional over the course of the study (Figure 8*B–D*).

## Discussion

In this study we showed that the HMG-binding lectibody AvFc shows broad genotype-independent anti-HCV activity. In addition, systemic administration of AvFc effectively protected chimeric human-mouse liver mice from infection with a genotype 1a virus without apparent toxicity, providing in vivo proof-of-concept for the lectibody’s antiviral potential.

The mechanism of HCV neutralization by AvFc likely is through binding to HMGs on the E1/E2 envelope protein dimer, which blocks their interaction with host cell receptors and viral entry. Unlike HIV envelope glycoproteins, whose glycan content can vary widely between strains, the number and position of glycosylation sites on E1/E2 are highly conserved, indicating their critical role in HCV’s infectious processes.^27^ The notion that AvFc functions as an entry inhibitor is supported by the fact that the lectibody has affinity to the E2 protein^24^ and that other mannose-binding lectins, such as Griffithsin or Cyanovirin-N, inhibit entry in this manner.^28^^,^^29^ AvFc inhibited multiple genotypes of HCV with average 50% inhibitory concentrations more than 100-fold lower than that of the monomer Avaren lectin (Table 1), indicating that the multivalent recognition of HMGs on the surface of the virus, brought about by the dimerization of Avaren lectin via Fc fusion, led to greater entry inhibition. Unlike other antiviral lectins, however, the inclusion of the human IgG1 Fc region implicates the possibility of Fc-mediated effector functions, such as antibody-dependent cell-mediated cytotoxicity, against infected cells. In fact, Fc-mediated effector functions greatly contributed to the antiviral potency of AvFc against HIV, as determined by a primary cell-based inhibition assay and an antibody-dependent cell-mediated viral inhibition assay.^24^ Accordingly, the remarkable efficacy seen in the present in vivo HCV challenge study may be partially Fc-mediated. Further investigations are necessary to address this possibility.

The present study also showed that AvFc therapy is well tolerated in mice and human hepatocytes because every-other-day intraperitoneal administration of 25 mg/kg AvFc, of up to 11 doses, did not show any obvious toxicity in PXB mice by gross necropsy or histopathology of engrafted human hepatocytes, and it did not result in significant changes in body weight, h-Alb level, or ALT level (Figures 6 and 7). This corroborates our previous observation that AvFc administration, both intraperitoneally and intravenously, was well tolerated and produced no toxicity in mice, rats, or rhesus macaques.^24^ We hypothesize that the lack of any significant toxicity is attributable to the unique HMG-binding mechanism of AvFc, whereby it requires multivalent interaction with several HMGs in proximity to show high-affinity binding to a glycoprotein target. In line with this hypothesis, Hoque et al^30^ showed that the 3 binding pockets of the parent lectin actinohivin can bind up to 3 independent HMGs, providing high-affinity binding when the HMGs are in relatively close proximity. This implies that AvFc may not interact effectively with healthy normal cells and tissues that do not usually show clusters of HMGs on their surfaces. In contrast, glycoproteins of many enveloped viruses show a high proportion of these immature forms of *N*-glycans.^20^, ^21^, ^22^ Although HCV E2 has fewer *N*-glycosylation sites (approximately 11) than the HIV gp120 (which has between 20 and 30, depending on the strain), E2 likely is present on the surface of HCV at a higher density and thus provides higher local concentrations of HMGs.^31^ Further studies are necessary to show a threshold HMG concentration that enables efficient interaction between AvFc and the surfaces of cells or viruses.

Although alcoholic liver disease has now surpassed HCV infection as the number one indication for liver transplantation in the United States, a large number of procedures will continue to be performed for the foreseeable future in patients with HCV-related decompensated cirrhosis.^32^ A major outstanding issue is the lack of effective treatment protecting the allograft liver from recurrent infection by the virus that remained circulating in the periphery at the time of transplant. As a consequence, re-infection of donor livers occurs universally, as early as in the first 90 minutes of reperfusion,^17^ and can result in accelerated fibrosis and increased risk of graft failure, cirrhosis, and hepatocellular carcinoma.^33^ In fact, allograft failure resulting from re-infection is the leading cause of secondary transplants and death in HCV-infected patients who have received a liver transplant.^34^ Patients cured of HCV with DAAs after liver transplantation still have a higher-than-normal risk of hepatocellular carcinoma,^35^ and the high cost of the drugs represents a significant barrier to their widespread use. Furthermore, emergent drug resistance, even in DAA combination therapies, although rare, represents a particular challenge for further treatment.^36^ Unlike DAAs, entry inhibitors neutralize circulating viruses and physically block the viral infection of target cells. The use of entry inhibitors perioperatively upon liver transplantation, either alone or in combination with DAAs, may improve treatment outcomes significantly.^34^^,^^37^ Thus, although the effectiveness of DAAs is not in question, there still are unmet needs that may be addressed through the use of entry inhibitors.

To date, no entry inhibitor has been approved for the treatment or prevention of HCV. Two major drug candidates, Civacir (Biotest AG, Dreieich, Germany) and MBL-HCV1 (MassBiologics, Mattapan, MA), have shown some promise in clinical trials (NCT01804829 and NCT01532908).^38^^,^^39^ Although larger studies are needed, it appears that entry inhibitors in combination with DAAs may represent a new treatment paradigm for HCV patients receiving a liver transplant. Despite both MBL-HCV1 and Civacir being capable of neutralizing a broad range of HCV genotypes, viral resistance still can develop through mutations in the envelope proteins E1/E2, in particular through shifting glycan positions.^40^^,^^41^ In this regard, AvFc in its own right could be less susceptible to amino acid mutations because it targets the glycan shield of the virus rather than a specific epitope. Deletions of glycans, even if occurring after prolonged exposure to a carbohydrate-binding agent such as AvFc, may result in a significant decrease in viral fitness by decreasing E1/E2 incorporation into HCV particles or increased susceptibility to humoral immunity resulting from a breach in the glycan shield.^27^^,^^42^ Our results provide a foundation to test the earlier-described hypotheses and feasibility of the HMG-targeting anti-HCV strategy. Of note, a unique advantage of AvFc over the 2 antibody-based entry inhibitor candidates described earlier is that the lectibody has the capacity to neutralize both HIV^24^ and HCV (present study). Accordingly, AvFc may provide an effective means (eg, pre-exposure prophylaxis) to protect high-risk populations against HIV/HCV co-infection, such as health care workers and injection drug users.^43^^,^^44^

In conclusion, the present study provides an important proof of concept for the therapeutic potential of AvFc against HCV infection via targeting envelope HMGs. In particular, the lectibody may provide a safe and efficacious means to prevent recurrent infection on liver transplantation in HCV-related end-stage liver disease patients. Other potential utilities of AvFc may be found in pre-exposure prophylaxis against HIV/HCV co-infection in high-risk populations, as well as in the context of transplantation of organs from HCV-infected donors to HCV-negative recipients, which may help alleviate the severe shortage of donor organs available for transplantation.^45^^,^^46^ Further studies are warranted to determine a dose-response relationship, therapeutic window, and feasibility of intravenous or subcutaneous dosing routes, as well as to assess the efficacy of AvFc against established infection.

## Materials and Methods

### Animal Care

The use of animals was approved by the University of Louisville**’**s Institutional Animal Care and Use Committee and the Animal Ethics Committee of PhoenixBio Company, Ltd (resolution 2281). All animals were given a standard diet and water ad libitum and were housed in a temperature- and humidity-controlled facility with a 12-hour day/night cycle.

### Production of AvFc and Non–HMG-Binding AvFc Variant

AvFc and AvFc^lec-^ were produced by agroinfiltration with magnICON (Icon Genetics GmbH, Halle, Germany) vectors in *Nicotiana benthamiana* plants as previously described.^24^ AvFc was purified from plants after a 7-day incubation period using protein A and ceramic hydroxyapatite chromatography.

### HCV Neutralization Assays

Huh-7 cells^47^ and HEK-293T cells (American Type Culture Collection, Manassas, VA) were cultured in Dulbecco's modified Eagle medium supplemented with 10% heat-inactivated fetal calf serum and 1% penicillin/streptomycin. To produce HCVcc, we used a modified version of the plasmid encoding JFH1 genome (genotype 2a), provided by T. Wakita (National Institute of Infectious Diseases, Tokyo, Japan).^48^^,^^49^ The H77/JFH1 chimera, which expresses the core-NS2 segment of the genotype 1a polyprotein within a genotype 2a background, has been described previously.^50^ The genotype 4a ED43/JFH1,^51^ genotype 5a SA13/JFH1,^52^ and genotype 6a HK6a/JFH1^53^ infectious HCV recombinants were provided by J. Bukh (University of Copenhagen, Copenhagen, Denmark). Retroviral pseudotypes bearing HCV envelope glycoproteins of JFH1 virus (HCVpp) expressing the *Firefly* luciferase reporter gene were produced in HEK-293T as previously described.^54^ Inhibitory effects were determined by quantifying infectivity by indirect immunofluorescence with the anti-E1 monoclonal antibody A4^55^ or an anti-NS5A polyclonal antibody kindly provided by M. Harris (University of Leeds, Leeds, UK).

### Formulation Buffer Optimization

Initial buffer screening was performed in 30 mmol/L glutamate, acetate, citrate, succinate, histidine, and phosphate buffers at pH 4.5–7.5 (Table 2). All the buffer agents were purchased from MilliporeSigma (Burlington, MA). AvFc was diafiltrated and adjusted to 1 mg/mL (or 62.5 μmol/L) in respective buffers. Stability was evaluated by sodium dodecyl sulfate–polyacrylamide gel electrophoresis after incubation for 2 weeks at 37°C. The melting temperatures of AvFc were determined by differential scanning fluorimetry performed on an Applied Biosystems StepOnePlus reverse-transcription polymerase chain reaction (RT-PCR) system as described previously.^24^ Briefly, AvFc formulated in various buffers at a concentration of 50 μmol/L was mixed with a final concentration of 50× SYPRO Orange (S6651; ThermoFisher Scientific) in a 96-well template. The melting temperature was determined by the vertex of the first derivative of the relative fluorescence unit values in the melt curves. AvFc formulated into the optimized histidine buffer or PBS then was concentrated to 10 mg/mL and incubated at 4°C or room temperature. Absorbance at 280 nm and 600 nm was measured immediately after concentration and then again after 16 and 72 hours. A_280_ was measured after centrifugation of precipitate.

### Pharmacokinetic Analysis

A pharmacokinetic profile for AvFc was generated after a single 25-mg/kg intraperitoneal injection in C57bl/6 mice (The Jackson Laboratory, Bar Harbor, ME) (8-week-old males and females; n = 4 per time point) and sampling blood at 0.5, 1, 2, 4, 8, 12, 24, and 48 hours after injection. The concentration of AvFc then was measured using an HIV gp120-coated enzyme-linked immunosorbent assay. Briefly, a recombinant gp120 (HIV CM235; AIDS Reagent Program; National Institutes of Health, Bethesda, MD) was coated overnight at 0.3 μg/mL followed by blocking with 5% dry milk-in PBS, pH 7.4, 0.05% Tween 20 (PBST). Serum samples at varying dilutions were incubated for 2 hours, followed by detection by a goat anti-human Fc–horseradish-peroxidase secondary antibody (ThermoFisher Scientific). The plasma concentration of AvFc was calculated by interpolating from a standard curve. PK parameters were calculated using the PKSolver Microsoft Excel add-on.^56^

### Toxicologic Analysis and HCV Challenge Study in PXB Mice

The mouse model of toxicologic analysis and HCV infection and toxicologic analysis was performed in PXB mice (complementary DNA–uPA^wild/+^/SCID, complementary DNA–uPA^wild/+^: B6; 129SvEv-Plau, SCID: C.B-17/Icr-*scid/scid* Jcl; reviewed by Tateno and Kojima^25^). These mice contain transplanted human hepatocytes with a replacement index of greater than 70% as determined by blood h-Alb measurements before virus inoculation.^57^ Blood h-Alb levels indicate the level and integrity of human hepatocyte engraftment in the mouse liver. Mice were separated into the following 3 treatment groups: AvFc^lec-^ (25 mg/kg, n = 5) for 11 doses, or AvFc (25 mg/kg, n = 5 each) for 8 or 11 doses. The initial treatment was co-administered intraperitoneally with virus inoculation (5 × 10^5^ copies/kg) on day 0 with a genotype 1a strain (PBC002), and treatment continued every other day thereafter. The general conditions and body weights of the animals were monitored every other day, while serum HCV RNA and blood h-Alb were measured every 7 days by RT-PCR or latex agglutination immunonephelometry (LZ Test Eiken U-ALB; Eiken Chemical Co, Ltd, Tokyo, Japan), respectively. The HCV RNA RT-PCR assay was developed based on the method described by Takeuchi et al^58^ with modifications, and validated by PhoenixBio for use in this animal model. The lower limit of quantification was determined to be 4.0 × 10^4^ copies/mL. Serum ALT 1 levels were determined either using a Fujifilm DRI-CHEM NX500sV clinical chemistry instrument (Fujifilm, Tokyo, Japan) or by enzyme-linked immunosorbent assay (Institute of Immunology Co, Ltd, Tokyo, Japan). At study termination on day 35, animals were killed and subject to gross necropsy. Blood also was drawn via cardiac puncture and used for ALT, HCV RNA, and h-Alb analyses.

### Histopathologic Analysis of Liver Tissues

H&E-stained liver sections from 3 to 4 mice per group were generated by Nara Pathology Research Institute Co, Ltd (Nara, Japan) and evaluated by pathologists at SkyPatho, LLC (Ube, Japan). All slides were examined by a blinded, board-certified veterinary pathologist under a light microscope (BX43; Olympus Corporation, Tokyo, Japan). The tissues were assigned a severity score for a number of characteristics based on the 5-point scoring system of the Clinical Data Interchange Standards Consortium's Standard for Exchange of Non-clinical Data (CDISC SEND) Controlled Terminology, as follows: 0, unremarkable; 1, minimal; 2, mild; 3, moderate; 4, marked; 5, severe; and P, present.

### Statistical Analyses and Data Analysis

Statistical significance was analyzed by GraphPad Prism 6 software (La Jolla, CA). Mouse body weights, albumin, ALT, and HCV RNA levels were compared using a repeated-measures 2-way analysis of variance with the Geisser–Greenhouse correction. Multiple comparisons between groups at each time point were conducted and corrected using the Tukey method with the threshold of significance set at *P* = .05. Liver:body-weight ratios were compared using 1-way analysis of variance. All authors had access to the study data, and reviewed and approved the final manuscript.
