# Supplementary material for: Safety and Efficacy of Avaren-Fc Lectibody Targeting HCV High-Mannose Glycans in a Human Liver Chimeric Mouse Model
Source: Cell Mol Gastroenterol Hepatol. 2020 Aug 27;11(1):185–98. doi: 10.1016/j.jcmgh.2020.08.009 (PMC7451001; doi:10.1016/j.jcmgh.2020.08.009)
Supplement: Supplementary Material [file mmc1.pdf]

SUPPLEMENTAL MATERIAL

Safety and Efficacy of Avaren-Fc Lectibody Targeting HCV High-Mannose Glycans in a Human Liver Chimeric Mouse Model

Matthew Dent[a], Krystal Hamorsky[b,c,d], Thibaut Vausselin[e], Jean Dubuisson[e], Yoshinari Miyata[f], Yoshio Morikawa[f], Nobuyuki Matoba[a,c,d,*]

[a]Department of Pharmacology and Toxicology, University of Louisville School of Medicine, Louisville, KY, USA

[b]Department of Medicine, University of Louisville School of Medicine, Louisville, KY, USA

[c]James Graham Brown Cancer Center, University of Louisville School of Medicine, Louisville, KY, USA

[d]Center for Predictive Medicine, University of Louisville School of Medicine, Louisville, KY, USA

[e]University of Lille, CNRS, INSERM, CHU Lille, Institut Pasteur de Lille, U1019 – UMR 8204 – CIIL – Center for Infection & Immunity of Lille, Lille, France

[f]PhoenixBio USA Corporation, New York, NY, USA

Running Head: HCV Inhibition of a Lectin-Fc Fusion Protein

*Address correspondence to Dr. Nobuyuki Matoba, n.matoba@louisville.edu

Pathology Report

Histopathological Evaluation of the Liver from PXB-mice

Sponsor’s Study No.: PBC-HI18-006

SkyPatho Contract No.: 2018-PBC-08

SkyPatho, LLC

Study Title:	Histopathological evaluation of the liver PXB-mice
Sponsor' Study No.:	PBC-HI18-006
Sponsor:	PhoenixBio Co., Ltd.<br>3-4-1, Kagamiyama, Higashi-Hiroshima, 739-0046, Japan
Sponsor's Representative:	Masakazu Kakuni, DVM, PhD<br>Tel: +81-82-431-0016<br>Fax: +81-82-431-0017
Study Facility:	SkyPatho, LLC<br>5-2-11, Kame-ura, Ube, Yamaguchi, 755-0002, Japan<br>Tel/Fax: +81-836-39-5553
Contract No.:	2018-PBC-08
Study Pathologist:	Katsumi Takaba, DVM, PhD, DJCVP, DJSTP, FIATP
Study Duration:	Start of study (receipt date of the shipment slides): November 2, 2018<br>Completion of study (definitive date of this report): November 29, 2018

Signature: Katsumi Takaba   29. Nov., 2018

Katsumi Takaba   Date

Study Pathologist

1. OBJECTIVE

The objective of this study was to histopathologically evaluate the liver removed from the PXB-mice that were treated with test compound.

2. MATERIALS AND METHODS

2.1 Materials

The following specimens provided from PhoenixBio Co., Ltd. were used as materials of this study.

Nara Pathology Research Institute Co., Ltd. (Nara, Japan) produced the Hematoxylin and Eosin (H&E) staining slides of the livers from the male PXB-mice (Donor of hepatocytes: JFC). The slides were shipped from Nara Pathology Research Institute Co., Ltd. and received on November 2, 2018.

The group compositions and dose levels of this study were as follows:

Group No.	Test compound (Dose)	Frequency*1	No. of mice (mouse ID)*3
1	Vehicle	Days 0*2, 2, 4, 6, 8, 10, 12, 14, 16, 18 & 20	3 (101~103)
2	Control (25 mg/kg)	Days 0*2, 2, 4, 6, 8, 10, 12, 14, 16, 18 & 20	4 (201~204)
3	AvFc (25 mg/kg)	Days 0*2, 2, 4, 6, 8, 10, 12 & 14	4 (301~304)
4	AvFc (25 mg/kg)	Days 0*2, 2, 4, 6, 8, 10, 12, 14, 16, 18 & 20	4 (401~404)

*1: Each test compound was intraperitoneally administered (q2d, Dosing volume: 10 mL/kg).
*2: Mixture of the test compound and the inoculum (PBC002) was intraperitoneally administered (q1d)
*3: All animals were sacrificed on Day 42 (The age on Day 0 was 16-17 weeks old).

2.2 Methods

All slides were blindly examined under a light microscope (BX43, Olympus Corporation, Tokyo, Japan) by a board-certificated veterinary pathologist. The severity grades were classified as 0: unremarkable, 1: minimal, 2: mild, 3: moderate, 4: marked and 5: severe, based on 5-point scoring system of the CDISC SEND Controlled Terminology (updated on June 29, 2018).

Statistical analysis was not performed.

3. RESULTS

The results are presented in Figures 1 to 9 and Table 1.

In the mouse hepatocyte area, unremarkable changes were detected in all 4 groups.

In the human hepatocyte area, slight to moderate severity of macrovesicular fatty change, as a characteristic change of human hepatocytes in the PXB-mouse, was observed in all mice of all 4 groups (Figures 1-3). Minimal severity of inflammatory cell infiltration around vacuolated hepatocytes (Figures

4-5) were observed in a few mice throughout Groups 2 to 4.

As for other changes, in Group 1, hepatocellular carcinoma (Figures 6-7) and osseous metaplasia (Figure 8) were observed in one mouse, respectively. In Group 4, pigmentation in the Glisson’s sheath (Figure 9) was observed in one mouse.

4. DISUCUSSION AND CONCLUSION

The liver slides taken from PXB-mice administered test compound were histopathologically examined to evaluate its effects.

In all mice in this study, as a characteristic change of human hepatocytes in the PXB-mouse, macrovesicular fatty change (vacuolation) was observed. Hepatocellular carcinoma and osseous metaplasia were judged to be incidental lesions because they were only detected in Group 1. Inflammatory cell infiltration around vacuolated hepatocytes was considered not to be induced by treatment of the test compound, because this change was occasionally noted in intact PXB-mice. Pigmentation in the Glisson’s sheath was also considered not to be induced by treatment of the test compound, because this change was focal and occasionally noted in many mouse strains as incidental lesions.

In conclusion, adverse effects on the liver of PXB-mouse due to administration of the test compound were not confirmed in this study.

5. FIGURES

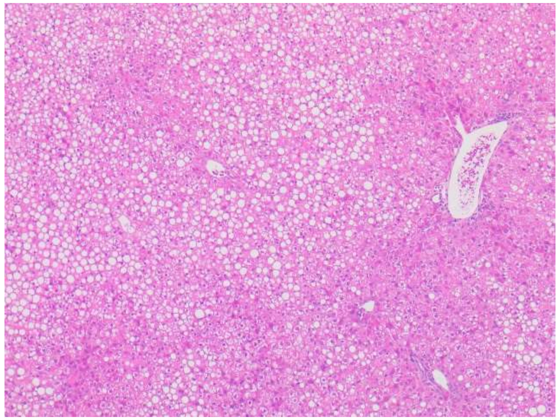

Figure 1; Animal No.: 103, H&E staining, x4
Low magnification of the liver of a PXB-mouse treated with Vehicle.

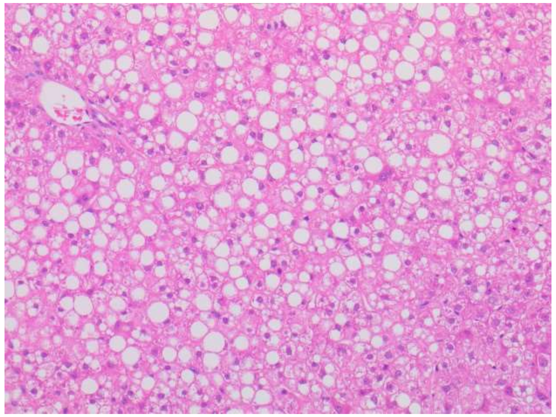

Figure 2; Animal No.: 103, H&E staining, x10
Shows a part of Figure 1. Many human hepatocytes contain a large well-defined single rounded vacuole within each cell (macrovesicular fatty change), characteristically.

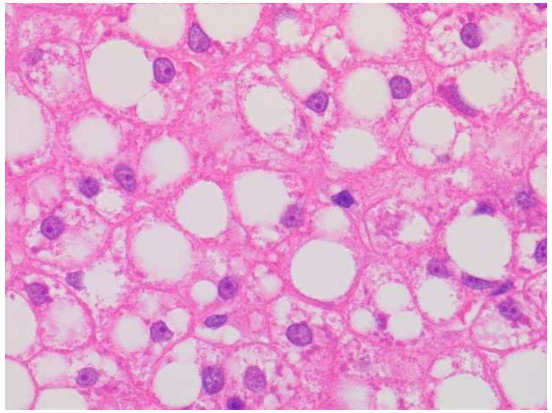

Figure 3; Animal No.: 103, H&E staining, x40
Higher magnification of Figure 2.

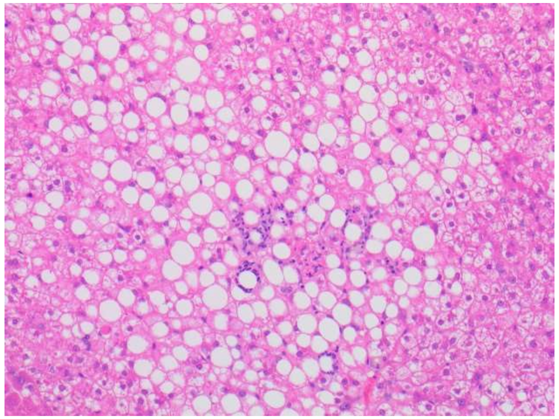

Figure 4; Animal No.: 202, H&E staining, x10
Small foci of Inflammatory cell infiltration are detected in the area of human hepatocytes in the liver of a PXB-mouse treated with Control.

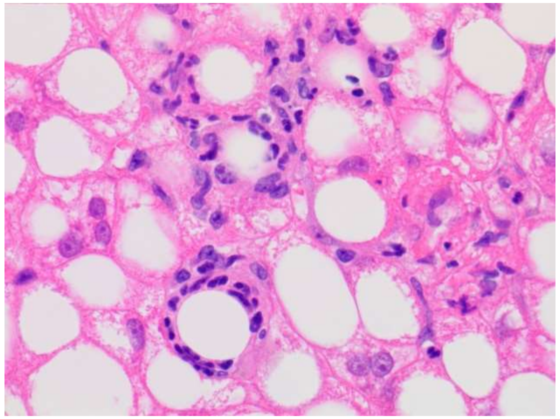

Figure 5; Animal No.: 202, H&E staining, x40
Higher magnification of Figure 4. Inflammatory cells are recognized to surround vacuolated human hepatocytes.

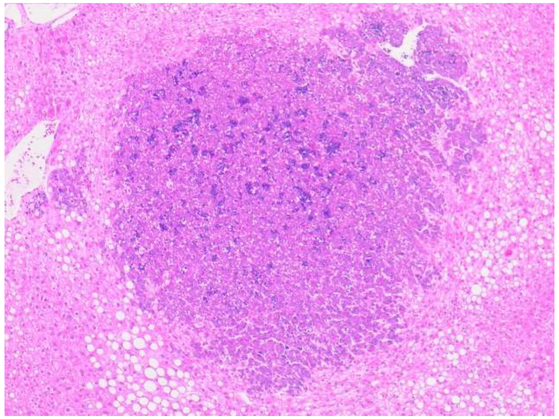

Figure 6; Animal No.: 101, H&E staining, x4
Hepatocellular carcinoma in the liver of a PXB-mouse treated with Vehicle.
.

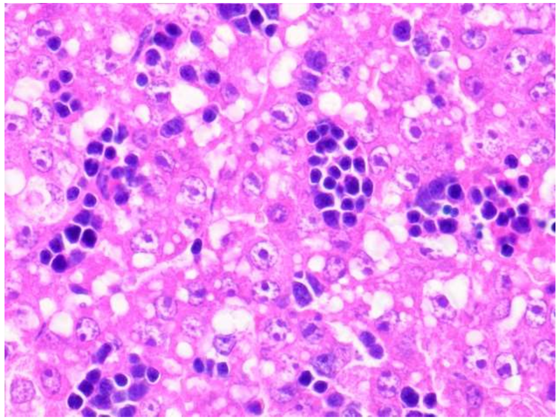

Figure 7; Animal No.: 101, H&E staining, x40
Higher magnification of Figure 6. Tumor cells are small and basophilic with cellular atypia. Foci of erythroblastic extramedullary hematopoiesis are present in the sinusoid.

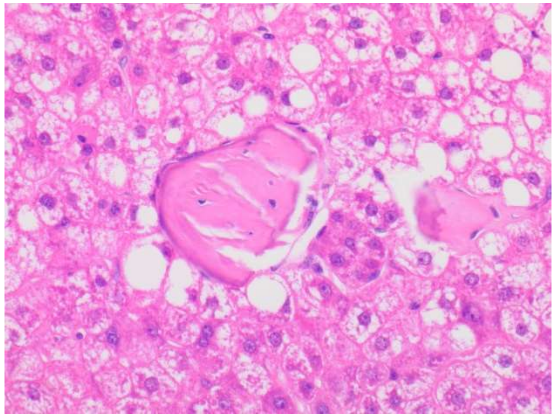

Figure 8; Animal No.: 102, H&E staining, x20
Osseous metaplasia in a PXB-mouse treated with Vehicle.

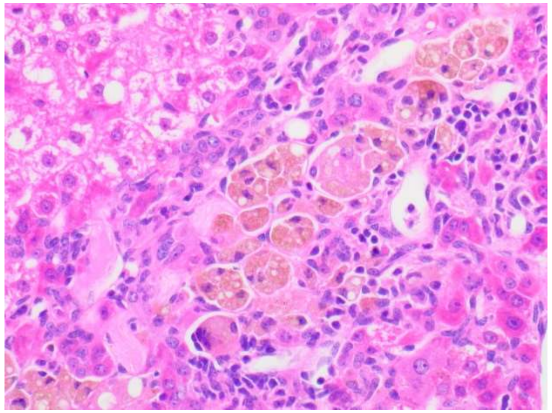

Figure 9; Animal No.: 401, H&E staining, x20
Histiocytic brown pigmentation in the Glisson's sheath in a PXB-mouse treated with AvFc2.

6. TABLE

6.1 Table 1

Area/Region Test Compound (mg/kg)	Vehicle			Control (25 mg/kg)				AvFc (25 mg/kg)				AvFc (25 mg/kg)			
Findings (comment) Animal No.	101	102	103	201	202	203	204	301	302	303	304	401	402	403	404
Area of mouse hepatocyte	0	0	0	0	0	0	0	0	0	0	0	0	0	0	0
Area of Human hepatocyte															
Fatty change, macrovesicular, hepatocyte	2	3	3	3	3	3	3	3	3	3	3	3	3	3	3
Infiltrate, inflammatory cell, around vacuolated hepatocyte	0	0	0	0	1	0	0	0	1	0	0	0	0	1	0
Portal Canal and Others															
Hepatocellular carcinoma, trabecular, with extramedullary hematopoiesis	P	0	0	0	0	0	0	0	0	0	0	0	0	0	0
Metaplasia, osseous	0	2	0	0	0	0	0	0	0	0	0	0	0	0	0
Pigmentation, brown, histiocyte, Glisson's sheath, focal	0	0	0	0	0	0	0	0	0	0	0	1	0	0	0

Severity system: 0: unremarkable, 1: minimal, 2: mild, 3: moderate, 4: marked, and 5: severe.
